# Review of Organism Density and Bacteriologic Conversion of Sputum among Tuberculosis Patients

**DOI:** 10.1155/2017/7052583

**Published:** 2017-07-11

**Authors:** Francis Adjei Osei, Anthony Enimil, Daniel Ansong, Dennis Odai Laryea, Nicholas Karikari Mensah, Evans Xorse Amuzu, Ebenezer Opambour Agyemang, Phans Oduro Sarpong, Isaac Nyanor, Denis Dekugmen Yar

**Affiliations:** ^1^Public Health Unit, Komfo Anokye Teaching Hospital, Kumasi, Ghana; ^2^Research and Development Unit, Komfo Anokye Teaching Hospital, Kumasi, Ghana; ^3^Department of Child Health, Komfo Anokye Teaching Hospital, Kumasi, Ghana; ^4^Kumasi Centre for Collaborative Research in Tropical Medicine, Kwame Nkrumah University of Science and Technology, Kumasi, Ghana

## Abstract

**Objective:**

This study sought to describe the trend of sputum organism density and the rate of bacteriological conversion among smear positive TB patients assessing care at the Komfo Anokye Teaching Hospital (KATH), Kumasi, Ghana.

**Methods:**

We conducted a retrospective patient folder review from January 2013 to March 2016 at the KATH, a tertiary hospital in Ghana. The data was entered into Microsoft Access database and exported into STATA for analysis. We applied basic descriptive statistics to study variables. Sputum conversion rate (SCR) was estimated using the number of negative tests recorded over a period (numerator) and the number of patients reported in the same period (denominator) and expressed as a percentage.

**Results:**

A total of 278 patient records with sputum smear positive at onset were studied. Before treatment sputum density detected in smear microscopy was as follows: 1 acid-fast bacillus (+) (*n* = 114), scanty (*n* = 19), ++ (*n* = 67), and +++ (*n* = 78). We recorded sputum conversion rate of 80.90%, 94.56%, and 98.31% in the intensive, continuation, and completion phases, respectively.

**Conclusion:**

This study has shown an increasing trend in sputum conversion of smear positive pulmonary tuberculosis and an increasing trend in loss to follow-ups among tuberculosis patients on treatment.

## 1. Background

Tuberculosis (TB) is an airborne disease caused by the bacterium* Mycobacterium tuberculosis*. In 2014, 9.6 million people were diagnosed with TB, with 5% developing multidrug resistant TB and a mortality of 15.6%. 10.4% of the affected were children, and 12.5% also had HIV [1]. TB was declared an emergency in the African region in 2005 when its annual incidence quadrupled in most African countries from the 1990 incidence and continues to rise across the continent killing by more than half a million each year [2]. The disease incidence was reported in Ghana as 66 cases per 100,000 in 2013 [3]. In 2014, the incidence was reported as 165 with a prevalence of 282 per 100,000 [1].

In developing countries, sputum smear microscopy is the primary tool for detecting pulmonary tuberculosis (PTB) [4]. Smear positive TB is inferred when a suspected patient presents with at least 2 sputum specimens positive for acid-fast bacilli (AFB) which are laboratory confirmed by Ziehl-Neelsen method. This method is used to confirm that the acid-fast bacteria are* Mycobacterium tuberculosis.* Numbers of bacteria are recorded as +++ (when there are presence of more than 10 AFB/field in at least 20 fields), ++ (when 1–10 AFB/field are present in at least 50 fields), + (when 10–99 AFB/field are seen at least in 100 fields), or exact number when 1–9 AFB/100 fields are seen [5]. The contagiousness of smear positive TB cases is dependent on organism density as well as the rate of conversion with unfavourable outcomes such as death [6].

Confirmed TB cases undergo the Direct Observed Therapy Short-Course (DOTS) treatment regimen for six months, divided into two phases: a 2-month intensive phase and a 4-month continuation phase of treatment. If the patient does not get cured after 6 months of treatment, they are then enrolled on an 8-month retreatment regimen.

During treatment the smear microscopic examination is repeated for a minimum of 3 times (at 2-3 months of treatment, at 5 months after treatment, and at the end of treatment) to monitor organism density clearance over time and also promote adherence to treatment. Previous studies reviewed show that the density of tuberculosis bacteria reduces with treatment continuations. The evidence points to sputum conversion rate of 80%–93% in the intensive phase [6–9] with progressions through the continuation phase to the completion phase at the end of treatment. The treatment success rate in Ghana is currently estimated at 85% [10]. Nonadherence to TB drug could lead to relapse and could eventually lead to drug resistance (polydrug resistance, multidrug resistance, and extensive drug resistance) [11].

There is however inadequate evidence on sputum organism density levels among smear positive TB patients and sputum conversion rates (SCR) across the treatment phases in Ghana. The current study however aimed at describing the trend of sputum organism density and bacteriological conversion among smear positive TB before treatment through to the end of treatment in patients assessing care at the Komfo Anokye Teaching Hospital (KATH), Kumasi, Ghana.

## 2. Materials and Method

### 2.1. Study Design

A retrospective cross-sectional study was designed to review 283 medical records of smear positive tuberculosis patients reporting to the KATH. All patients who underwent screening for sputum smear microscopy at the KATH through the study periods were included in the study.

### 2.2. Study Site

Komfo Anokye Teaching Hospital (KATH) is the second largest hospital in Ghana located in Ashanti Region of Ghana. It serves a population of over 4 million within and outside Ashanti Region. It is the main referral centre for the middle through to the northern zone of Ghana.

The TB department of the hospital is a collaboration of the Public Health Unit, the Medicine Directorate, and the Diagnostics Directorate. The department is responsible for counselling and testing suspected cases of tuberculosis and providing treatment. The sputum smear microscopy services of the laboratory are also extended to other facilities outside the hospitals.

The study included persons who had been screened for TB and were tested positive to sputum smear microscopic examination.

### 2.3. Data Collection and Analysis

The national TB control program has a standard register termed as the “TB register” for recording all relevant information on clients who assess the services. The TB register contains information on the personal characteristics of the clients, details of laboratory information, treatment, and the treatment outcomes.

A systematic review of records of patients aged 18 years and above who presented with smear positive tuberculosis between January 2013 and March 2016 was conducted. This period was chosen based on the reliability and availability of medical records for the study. All information contained in the TB register was entered into a Microsoft Access 2010 database and exported into STATA SE 12.0 software (Stata Corp LP, USA) for analysis. Variables were described using basic descriptive statistics and numerical variables were analyzed by measures of central tendencies and dispersions whereas categories were reported in proportions. Sputum conversion rate (SCR) was estimated by dividing the number of negative results at each monitoring time point by the total number of patients reported and expressed in percentages. Patients who were lost to follow-up were excluded from the SCR estimation. The study however did not estimate the culture conversion since the facility did not exist at the study site.

### 2.4. Ethical Consideration

Permission to publish the data on human subjects was sought from the joint Ethical Review Board of the Komfo Anokye Teaching Hospital and Kwame Nkrumah University of Science and Technology which is the Committee on Human Research, Publications and Ethics (CHRPE).

## 3. Results

A total of 278 cases of smear positive TB patients reported to the Komfo Anokye Teaching Hospital TB department out of 1403 adult screens. On average, about 7 cases of smear positive TB cases were detected monthly over the 38 months' study period (January 2013 to March 2016).

The median age of smear positive tuberculosis cases was 38 years (IQR 30–48 years). Half (50.00%) of the patients were aged 31–50 years. There were more males (*n* = 162, 58.27%) than females (*n* = 116, 41.73%). Also, about eighteen percent (18.35%, *n* = 51/273) tested positive to HIV at baseline (Table 1).

All participants had positive acid-fast microscopy with various densities of bacteria visualized. 114 had + AFB detected in sputum smear microscopy and scanty AFB was detected in 19 (6.83%) cases. At the first monitoring check between the 2nd and 3rd months of the 6-month treatment by DOTS, 80.9% (*n* = 144/178) of the cases put on treatment had no organism in sputum detected and about 20% tested positive. The study further observed that at the next monitoring test at month 5 of treatment almost 95% (*n* = 139/147) tested negative to sputum smear microscopy and 5.44% (*n* = 8/147) tested positive. At the last laboratory investigation done at end of the treatment period, 98.31% of patients tested negative while 2 (1.69%) still remained positive with one having as high as +++ AFB counts. The number of positive cases (of all bacteria counts) reduced with time and sputum conversion rate of 80.90%, 94.56%, and 98.31% in the intensive phase (2-3 months of treatment), continuation phase (5 months after treatment follow-up), and completion phase, respectively (Table 2).

Out of all patients who were smear positive at start of treatment, more than half (57.55%, *n* = 160/278) were lost to follow-up at the end of the 6-month treatment period. This number comprises patients who had tested negative during the course of the study and those who still had significant sputum organism densities.

## 4. Discussion

Smear positivity and bacteriological density in TB treatment clinics in Sub-Saharan Africa are key public health information required to address individual treatment progress and public risk that patients pose to the community. In the study an average of 7 sputum smear positive cases reported monthly to KATH suggests a high burden of the disease in the study setting. This finding which has similarities with other reports from developing countries [12] has negative clinical and public health implications.

The prevalence of TB in the active working age group (18–60 years) makes it a matter of public concern. Persons in this age category spread the disease much more than other age groups because of their contacts at workplaces. This calls for consideration to roll out community based surveillance targeting various workplace settings in search of signs and symptoms of tuberculosis. The fact that there are more males than females with the disease is consistent with other studies [13–17].

All patients undergoing treatment of smear positive microscopy should be monitored to assess their response to the treatment. As recommended by WHO, new pulmonary TB patients must go through sputum smear microscopy at the end of the two-month initial phase of treatment and if the smear is positive, then it is continued on the initial phase for an extra month before proceeding with the standard four- or six-month continuation phase [18]. In our study, we observed sputum conversion rate of about 80% when the smear microscopic investigation was conducted at 2 months after treatment. This finding is consistent with some studies [7, 8] and can be considered to be of low conversion rate when compared with other studies with conversion rate ranging from 90 to 93% [6, 9]. Also in the same period, about one in every three patients seeking treatment was lost to follow-up (LTFU). This is significantly high compared to other studies with about 93% follow-up rate in the 2nd month after the initial treatment phase [7]. In a similar study, defaulting treatment or loss to follow-up was found to be strongly associated with nonconversion of positive sputum smears in patients with pulmonary tuberculosis at the end of two months of treatment [6]. The large number of patients who were found to be LTFU in our study calls for concern. The second month of reexamination is particularly important because studies have shown that patients who continuously show positive result at this stage have an increased risk of TB relapse [19]. Conversely, it has been established that nonconversion of positive smears after two months of treatment is one of the strongest predictors for treatment failure [6, 9, 20, 21].

Sputum microscopy needs to be performed at least once a month until 2 consecutive negative specimens are obtained for patients being treated for pulmonary TB [19]. The study site repeats microscopic examinations after five months and at the end of treatment. The 5-month monitoring (also referred to as the continuation phase) is intended to examine patient response to treatment. A positive smear result at this stage could indicate a number of situations such as possibility of drug resistant TB and hence may require a change in the TB drugs. In our study, two patients tested positive to sputum microscopic at the end of treatment follow-up. However, these patients had no record of comorbid features like HIV. This is worrying because these patients can be a source of spread in the community. Again, there is the need to identify whether their failure was due to multidrug resistance which is a major public health concern.

The study did not cover the assessment of sputum smear reversion. This measures the extent to which acid-fast smear becomes positive again after negative conversion during antituberculosis treatment. A recent study conducted in Taiwan showed that sputum reversion occurs in about 10% of smear positives undergoing treatment in endemic area [22]. This phenomenon is a sign of suboptimal treatment or poor adherence and could offer an opportunity to evaluate the TB treatment regimen as well as evaluating the STOP TB program. However, continuous measurement of the* Mycobacterium* organism density offers a valuable evidence to assess treatment adherence among patients undergoing treatment at the KATH.

This study was limited in scope regarding AFB culture and drug susceptibility testing. The lack of this information limits the ability to make inference into culture conversions and detecting multidrug resistance.

## 5. Conclusion

This study has shown an increasing trend in sputum conversion of smear positive pulmonary tuberculosis and a cumulative increase in loss to follow-ups among tuberculosis patients on treatment. This information is relevant because it offers a valuable basis to consider a review of the DOTS policy to enhance its effectiveness in the health care delivery system in Sub-Saharan Africa.

## Figures and Tables

**Table 1:** Distribution of patients by sex, age groupings, and HIV status.

Variable	Frequency (*N* = 278)	Percentage
*Age*		
18–30 years	80	28.78
31–50 years	139	50
51–70 years	50	17.99
71 years and above	9	3.24
Median (IQR)	38 (30, 48)	
*Sex*		
Male	162	58.27
Female	116	41.73
*HIV status*		
Positive	51	18.35
Negative	100	35.97
Not recorded	127	45.68

**Table 2:** Trend of smear positive results recorded before treatment through to the end of treatment.

Monitoring period	Sputum conversion results						
	Negative	Scanty	+	++	+++	^*∗*^SCR	^*∗∗*^LTFU
Monitoring before treatment *n* = 278	—	19	114	67	78	—	—
Monitoring at 2-3 months after treatment *n* = 278	144	10	20	1	3	80.90	100
Monitoring at 5 months after treatment *n* = 178	139	3	3	1	1	94.56	31
Monitoring at end of treatment *n* = 147	116	1	0	0	1	98.31	29

^*∗*^SCR = sputum conversion rate; ^*∗∗*^LTFU = loss to follow-up.
